# Mattis dementia rating scale (DRS) normative data for the brazilian middle-age and elderly populations

**DOI:** 10.1590/S1980-57642013DN74000004

**Published:** 2013

**Authors:** Maria Paula Foss, Viviane Amaral de Carvalho, Thais Helena Machado, Geraldo Cássio dos Reis, Vitor Tumas, Paulo Caramelli, Ricardo Nitrini, Cláudia Sellitto Porto

**Affiliations:** 1 Psicóloga do Setor de Distúrbios do Movimento e Neurologia Comportamental do Hospital das Clínicas da Faculdade de Medicina de Ribeirão Preto da USP, Doutora em Ciências Médicas (Neurociências) da FMRP-USP, Ribeirão Preto SP, Brazil.; 2 Psicóloga, Neurologia Cognitiva e do Comportamento da Faculdade de Medicina da Universidade Federal de Minas Gerais, Belo Horizonte MG, Brazil.; 3 Fonoaudióloga, Doutora em Linguística pela UFMG, Pesquisadora no Ambulatório de Neurologia Cognitiva e do Comportamento do Hospital das Clínicas da UFMG, Belo Horizonte MG, Brazil.; 4 Estatístico do Departamento de Neurociências e Ciências do Comportamento da FMRP-USP, Ribeirão Preto SP, Brazil.; 5 Médico, Faculdade de Medicina de Ribeirão Preto - FMRP-USP, Ribeirão Preto SP, Brazil.; 6 Professor do Departamento de Clínica Médica da Faculdade de Medicina da UFMG; Coordenador do Ambulatório de Neurologia Cognitiva e do Comportamento do Hospital das Clínicas da UFMG, Belo Horizonte MG, Brazil.; 7 Diretor da Disciplina de Neurologia da Faculdade de Medicina da Universidade de São Paulo, São Paulo SP, Brazil.; 8 Psicóloga do Grupo de Neurologia Cognitiva do Comportamento da Clínica Neurológica do Hospital das Clínicas da FMUSP, Doutora em Ciências pela FMUSP, São Paulo SP, Brazil.

**Keywords:** DRS, illiterates, elderly, schooling, neuropsychological assessment

## Abstract

**OBJECTIVE:**

To expand norms for the Mattis Dementia Rating Scale (DRS) for the Brazilian middle-age and elderly populations.

**METHODS:**

The DRS was administered to 502 individuals without cognitive deficits, 312 women and 190 men, aged 50 years or over and with educational level ranging from 0 to 13 years or more. The sample was composed of subjects who participated in other studies, from Caeté (Minas Gerais state), Ribeirão Preto (São Paulo state) and São Paulo (São Paulo state). Participants were divided into four schooling groups (illiterate, 1 to 4 years, 5 to 12 years and 13 years or more). The subjects were divided into four groups according to age (50 to 60, 61 to 70, 71 to 80, and 80 years or over).

**RESULTS:**

Normative data for DRS scores are expressed as percentile values. The group with lowest schooling and subjects older than 80 years had the worst scores.

**CONCLUSION:**

As expected, age and education were strongly correlated with DRS scores. Illiterates and older old individuals performed worse than the other groups. These data might help to improve the accuracy of the diagnosis of cognitive impairment and dementia in Brazilian middle-age and elderly populations.

## Abstract (pt)

**OBJETIVO:**

Expandir normas para o *Mattis Dementia Rating Scale* (MDRS) na população brasileira de meia idade e idosa.

**MÉTODOS:**

A MDRS foi aplicada em 502 indivíduos, 312 mulheres e 190 homens, com idade de 50 anos ou mais e escolaridade de 0 a 13 anos ou mais anos. A amostra foi composta de sujeitos que participaram de outros estudos: Caeté (Minas Gerais), Ribeirão Preto (São Paulo) e São Paulo (São Paulo). Participantes foram divididos em quatro grupos de escolaridade (analfabetos, 1 a 4 anos, 5 a 12 anos e 13 anos ou mais). Os sujeitos foram divididos em quatro grupos de idade (50 a 60, 61 a 70, 71 a 80 e acima de 80 anos).

**RESULTADOS:**

Dados normativos dos escores da MDRS são apresentados em percentis. O grupo com menos escolaridade e sujeitos acima de 80 anos apresentaram escores mais baixos.

**CONCLUSÃO:**

Como esperado, idade e escolaridade foram fortemente correlacionados aos escores da MDRS. Analfabetos e indivíduos muito idosos apresentaram pior desempenho que os outros grupos. Os dados podem ajudar a melhorar a acurácia para o diagnóstico de prejuízo cognitivo e demência na população brasileira de meia-idade e idosos.

## INTRODUCTION

The Mattis Dementia Rating Scale (DRS)^1^ was originally created for the diagnosis of Alzheimer's disease (AD), but it has also been utilized for early detection of dementia, differential diagnosis between AD and other dementias, and dementia staging.^2-6^ The scale tasks are grouped into five subscales, each evaluating different cognitive areas, namely, Attention, Initiation/Perseveration (I/P), Construction, Conceptualization and Memory.

In the Brazilian population, Porto et al.^7^ have demonstrated the value of the DRS in differentiating 41 mild AD patients from 60 cognitively healthy controls and have shown the importance of establishing norms for this scale. To analyze the effects of age and schooling on the performance of the tests, patients and controls were separated into three age groups and three levels of schooling. In this sample population, the effects of education were more evident than the effects of age. The cut-off for total score was 122, yielding 91.7% sensitivity and 87.8% specificity.

Foss et al.^8^ analyzed the influence of low schooling and illiteracy on DRS performance in a group of cognitively healthy elderly with a broad distribution of educational level. The influence of schooling was studied in 62 controls and the total sample was divided into five groups of schooling: illiterates; 4 years; 8 to 9 years; 11 to 12 years and 15 to 16 years. Education interfered with individual performance on the DRS and illiteracy was a determinant factor of lower DRS scores.

As the previous studies were based on small samples, the generalization of these normative data is very limited. Hence, the aim of the present study was to expand the norms for the Mattis Dementia Rating Scale (MDRS) for the Brazilian population.

## METHODS

Overall, 502 individuals participated in these study, consisting of 312 women and 190 men, aged 50 years to 93 years old and with a wide distribution of educational level attained. The sample comprised healthy individuals who participated in other studies conducted in Caeté (Minas Gerais state), Ribeirão Preto (São Paulo state) and São Paulo (São Paulo state). Exclusion and inclusion criteria were described in previous studies.^7-10^

In order to investigate the influence of schooling on DRS performance, individuals were divided into four schooling groups: illiterates, 1 to 4 years of schooling (sch 1), 5 to 12 years of schooling (sch 2), and an above 13 years of schooling (sch 3). To be classified as illiterate the subject must have never been to school and be unable to read the sentence "Close your eyes" from the Mini-Mental State Examination (MMSE). In order to analyze the interference of age on test performance, this variable was divided into four groups: Group 1 from 50 to 60 years, Group 2 from 61 to 70 years, Group 3 from 71 to 80 years and Group 4 over 80 years.

The DRS was applied to all subjects in a single individual session and in the order recommended by the author. It should be noted that DRS tasks are presented in a fixed order, and only the Attention tests are not grouped in a sequence, as they also serve as distractors for the Memory subscale. Within each subscale, the most difficult tests are presented in first and second place and, if performed well, subsequent items in the subscale are credited as being performed correctly. The advantage of this procedure is that it allows shortening of the total testing time for individuals whose cognitive function is better preserved.

The number of points credited for the correct response varies in accordance with the tasks and the total points in each subscale score provide a partial score for that subscale. The partial scores are Attention, 37 points; Initiation/Perseveration (I/P), 37 points; Construction, 6 points; Conceptualization, 39 points; and Memory, 25 points. The maximum total possible score is 144 points.

All participants signed a written informant consent form and the study was approved by the respective Research Ethics Committees of the Hospital das Clínicas of the University of São Paulo School of Medicine, the Hospital das Clínicas of University of São Paulo at Ribeirão Preto, and of the Federal University of Minas Gerais.

**Data analyses.** The statistical analyses were conducted with the SPSS statistical software package version 17.0 for Windows and p-values of 0.05 or less were considered statistically significant.

Demographic variables (age, education and gender) were correlated to DRS scores (subscales and total score) using Pearson's tests (r). Also, the demographic variables (age and education) were compared to DRS scores (subscales and total score) by the independent samples test for multiple comparisons (ANOVA) and Tukey's post hoc test. DRS scores were transformed into percentiles and z-scores and subsequently into T-scores.

## RESULTS

The demographic information of this study group is shown in Table 1. Mean educational level was 8.00 years (Standard Deviation (SD)=5.28), with a greater number of individuals having 1 to 4 years of schooling. In relation to age, the mean was 71.00 years (SD=8.70), with a greater number of subjects aged 70 to 80 years (Table 1). Females comprised 62% of the study group, with 38% males. The illiterates comprised 15 women and 10 men, aged 75.12±6.83 years.

**Table 1:** Demographic data (n=502).

		n	%
Education	Illiterates	25	4.98%
	1-4 years	190	37.65%
	5-12 years	176	35.06%
	>13 years	112	22.31%
Age (years)	50-59 years	46	9.16%
	60-69 years	165	32.87%
	70-80 years	231	46%
	>80 years	60	12%
Gender	Male	190	37.85%
	Female	312	62.15%

Correlation of DRS scores and demographic data are shown in Table 2. Age and education were more strongly related to DRS scores than gender. Nonetheless, in the Attention subscale a significant correlation between gender and DRS scores was observed, with women performing worse than men.

**Table 2:** Correlations of DRS subscales and total score with demographic variables.

	Age r	Gender r	Education r
	-0.16 **	-0.095*	0.44**
Initiation / Perseveration	-0.26**	0.074	0.46**
Construction	-0.15**	-0.059	0.37**
Conceptualization	-0.23**	-0.053	0.56**
Memory	-0.20**	0.010	0.36**
Total score	-0.28**	-0.017	0.59**

* p: 0.05;

** p: 0.01

Analyses of DRS scores according to educational level yielded significant differences on the Attention subscale (illiterates < sch1 < sch2 < sch3); Initiation/Perseveration subscale (illiterates < sch1 < sch2 < sch3); Construction (illiterates < sch1 < sch2 and sch3); Conceptualization subscale (illiterates < sch1 < sch2 < sch3); Memory subscale (illiterates < sch1 < sch2 < sch3); and in total score (illiterates < sch1 < sch2 < sch3). As expected, groups with higher education had better scores than the other groups.

The age groups showed significant differences in the DRS scores for the Attention subscale (group age 1 > group age 3, group age 1 > group age 4, and group age 2 > group age 4), for the Initiation/Perseveration subscale (group age 1 > group age 3, group age 1 > group age 4, and group age 2 > group age 4), for the Construction subscale (group age 1 > groups 3 and 4), for the Conceptualization (group age 1 > groups age 2, 3 and 4), for the Memory (groups age 1 and 2 > group age 4) subscales and for total DRS score (group age 1 > group age 3, group age 1 > group age 4, and group age 2 > group age 4). In conclusion, group age 1 (youngest individuals) had higher scores than all the remaining groups, with group age 4 (oldest individuals) showing the worst scores.

Since the main purpose of the study was to expand DRS normative data for Brazil, the amount of people in each group according to age and education was stratified according to their significance so as to represent the population and its clinical relevance for Brazilian standards (education standards). However, other analyses were performed yielding interesting results, such as significant differences in the total DRS score between ages (> 85 years < 66 to 70 years < 56 to 60 years), but no significant differences between 75 to 85 years and 61 to 65 years, suggesting more differences for age division decades, in accordance with our tables.

In relation to schooling, illiterates performed significantly worse than their literate counterparts. Additional differences were observed for 1 and 2 years < 3 and 4 years < 11 and 12 years < 15 and 16 years < 16+ years of schooling). There were no significant differences between the groups with 5 to 10 years of education.

Normative data for DRS scores in the Brazilian older population are expressed as percentile values in Tables 3 to 5 for persons with 1-4 years of schooling (for those with other educational levels, the data are given in Tables 6 to 15, available at www.demneuropsy.com.br. Mean scores for the whole sample (n=502) were 35.04±1.95 for the Attention subscale, 34.19±3.52for Initiation/Perseveration, 5.58±1.05for Construction, 33.98±4.83for Conceptualization, 22.89±2.65for Memory and, finally, 131.7±10.99for total score. However, in accordance with the large variation in age and education, the data are better represented by years of schooling and age.

**Table 3:** Percentiles for subjects with 1-4 years of schooling and 50 to 60 years of age (n=16).

Percentile	Attention	I/P	Construction	Conceptualization	Memory	Total
1	33	28	6	27	19	125
5	33	28	6	27	19	125
10	33.7	30.1	6	29.8	19	126.4
25	34.25	32	6	34	21.25	130.2
50	35	35	6	37	24	134
75	36	36	6	37	24.75	137.7
90	36	37	6	39	25	138.3
Mean	35	34	6	35.6	22.87	133.7
SD	0.93	2.6	0	3.24	2.125	4.38

**Table 5:** Percentiles for subjects with 1-4 years of schooling and 71 to 80 years of age ( n=107).

Percentile	Attention	I/P	Construction	Conceptualization	Memory	Total
1	27	21	1	17	17	101
5	31.7	25.85	3	22	19	108.85
10	33	27	3.7	24.7	20	117
25	34	30	5	29.25	22	123
50	35	33	6	32	23	129
75	36	36	6	35	25	134
90	37	37	6	37	25	138
Mean	34.82	32.75	5.46	31.79	23	127.8
SD	1.81	3.66	1.1	4.6	1.90	8.88

DRS scores decreased with age and in those individuals with fewer years of schooling. (Tables 3 to 14). Illiterates presented worse results than all the educated groups (p<0.001) and their results should therefore be separated from the others as part of these Brazilian norms (Table 15). Due to the small number of illiterates in this sample it was not possible to allocate them into age groups as was done for individuals with the other educational levels.

## DISCUSSION

Normative data for Brazilian elderly is necessary to characterize this population without neurological impairment and to avoid misdiagnoses. Previous studies with the DRS were conducted in Brazil^7,8^ on small normative samples. Notably, there are no robust normative data for the Mattis Dementia Rating Scale (MDRS) in the Brazilian population, justifying this study's objective of expanding preliminary data.

Age and education were correlated with DRS scores confirming the results of previous studies^7,8,11-13^ and the importance of these variables to these scores. These variables affected the performance on the DRS total score and all subscales, suggesting the adjustment of scores by age and education to improve diagnostic accuracy. Gender correlated only with scores on the Attention subscale with worse performances for women.

Monsch et al.^16^ also failed to encounter any significant differences in DRS scores for gender, but this was not true for age and education. Bank et al.^17^ obtained correlation of DRS total score with all demographic data, including gender, age, education and race, a variable not assessed in our study. Rilling et al.^18^ found correlation and shared variance of DRS scores with gender accounting for 3% of the variance, age (7%) and education (17%) among African Americans, emphasizing the higher effect of education on these scores. In the study by Pedraza et al.,^13^ age and education were significantly associated with DRS total score and subscales. Gender was associated with the Construction subscale. Mean Construction scores differed significantly for men and women only between the ages of 80 and 84.

In the study by Rosselli et al.,^14^ the effect of education and gender on total MMSE scores was analyzed using the "serial 7's" (attention) and the "backward spelling" (working memory) items. Women scored significantly lower than men on the MMSE "serial 7's". In another study employing the MMSE,^15^ women with less than 3 years of education scored significantly lower than men with the same educational level whereas no gender differences were observed in the groups with higher levels of schooling.

Education also accounted for significant differences between illiterate and literate subjects. In our study, individuals with higher education had better performance than all the other groups. Literacy is significantly associated with virtually all neuropsychological measures and the impact of literacy is reflected in different spheres of cognitive functioning. Learning to read reinforces and modifies certain fundamental abilities, such as verbal and visual memory, phonological awareness, and visuospatial and motor skills.^19^ Ostrosky-Solis et al.^20^ analyzed the performance of 64 illiterate normal subjects on neuropsychological tests and the largest educational effect was noted in constructional abilities, language, phonological verbal fluency and conceptual functions.

In a study conducted in Brazil with the DRS,^8^ 62 controls without neuropsychiatric disorders aged 65 to 75 years were distributed into five groups according to schooling level. The first group was composed of illiterates, followed by 4 years, 8 to 9 years, 11 to 12 years and 15 to 16 years of schooling. Illiterates had the worst scores compared with all the other literate groups whose scores rose with increased years of education. Illiteracy and low education were determinant factors for worse DRS scores, where this was also the case in the present study. For total DRS scores, a tendency toward stability was observed for 5 to 10 years of education.

Total and subscale scores showed differences in relation to age, with worse performances associated with older ages. For DRS total score, a tendency of difference in score between decades was observed. Bennett et al.^21^ showed, in a sample of 82 nondemented nursing home residents aged 80 to 99 with a mean educational level of 11 years, that a large percentage performed in the impaired range on the DRS, particularly on the Initiation/Perseveration and Conceptualization subscales and also on total score. In the study of Ostrosky-Solis et al.,^20^ the aging effect was noted in visuoperceptual and memory scores.

In conclusion, for the elderly Brazilian population, education represents a determinant factor for DRS scores. As the validity of a normative group will depend on the similarity between the examinee and the demographic features of the normative sample, it is preferable to present normative samples in relation to age and education rather than by only one of these variables. This is necessary since the Brazilian population has a large variation in socioeducational and demographic data.

The present normative data expands and integrates different studies with the DRS for the elderly Brazilian population. It is now necessary to include other regions of this large country to render this more representative of the entire middle-age and elderly populations. Also, further work should include more participants in the age ranges of 50 to 60 years and over 80 years, incrementing our findings. As evidenced, there are some limitations to our study and practitioners must therefore also rely on their clinical judgment when analyzing DRS results.

This work can contribute to research and clinical assessment of cognitive aging and also indirectly to dementia diagnosis. We believe these normative data will help avoid overdiagnosis and improve the accuracy of the diagnosis of dementia in Brazil.

## Figures and Tables

**Table 4:** Percentiles for subjects with 1-4 years of schooling and 61 to 70 years of age (n=57).

Percentile	Attention	I/P	Construction	Conceptualization	Memory	Total
1	29	19	0	16	14	95
5	29	24	3.55	25	17.65	104
10	31	29	4	27	20	117
25	33	31	5	29	21	124
50	34.5	35	6	32.5	23	129
75	36	36.25	6	36	24	135.25
90	36.9	37	6	38	25	138
Mean	34.1	33.36	5.36	32.26	22.58	127.9
SD	2.02	3.949	1.12	4.54	2.37	9.77

## References

[1] Mattis S (1988). Dementia Rating Scale. Professional Manual.

[2] Lukatela K, Cohen R, Kessler H (2000). Dementia Rating Scale performance: a comparison of vascular and
Alzheimer's dementia. J Clin Exper Neuropsychol.

[3] Paolo AM, Trostr AI, Glatt SL, Hubble JP, Koller WCJ (1995). Differentiation of the dementias of Alzheimer's and Parkinson's
disease with the dementia rating scale. J Geriatr Psychiatry Neurol.

[4] Paulsen JS, Butters N, Sadek JR (1995). Distinct cognitive profiles of cortical and subcortical dementia
in advanced illness. Neurology.

[5] Rascovsky K, Salmon DP, Ho GJ (1998). Cognitive profiles differ in autopsy-confirmed Lewy body variant
vs pure Alzheimer's disease. Arch Neurol.

[6] Salmon DP, Thomas RG, Pay MM (2002). Alzheimer's disease can be accurately diagnosed in very mildly
impaired individuals. Neurology.

[7] Porto CS, Fichman HC, Caramelli P, Bahia VS, Nitrini R (2003). Brazilian version of the Mattis Dementia Rating
Scale. Diagnosis of mild dementia in Alzheimer's Disease. Arq
Neuropsiquiatr.

[8] Foss MP, Valle F de A, Speciali JG (2005). Influence of education on the neuropsychological assessment of
the elderly: application and analysis of the results from the Mattis
Dementia Rating Scale (MDRS). Arq Neuropsiquiatr.

[9] Caramelli P, Barbosa MT, Sakurai E (2011). The Pietà Study. Epidemiological investigation on
successful brain aging in Caeté (MG), Brazil. Methods and baseline
cohort characteristics. Arq Neuropsiquiatr.

[10] Porto CS (2006). A escala de avaliação de demência (DRS) no
diagnóstico de comprometimento cognitivo leve e doença de
Alzheimer.

[11] Schmidt R, Freidl W, Fazekas F (1994). The Mattis Dementia Rating Scale: normative data from 1, 001
healthy volunteers. Neurology.

[12] Lucas JA, Ivnik RJ, Smith GE (1998). Normative data for the Mattis Dementia Rating
Scale. J Clin Exp Neuropsychol.

[13] Pedraza O, Lucas JA, Smith GE, Petersen R, Graff-Radford NR, Ivnik RJ (2010). Robust and Expanded Norms for the Dementia Rating
Scale. Arch Clin Neuropsychol.

[14] Rosselli M, Tappen R, Williams C, Salvatierra J (2006). The relation of education and gender on the attention items of
the Mini-Mental State Examination in Spanish speaking Hispanic
elders. Arch Clin Neuropsychol.

[15] Rosselli D, Ardila A, Pradella G (2000). El Examen Mental abreviado (Mini-Mental State Examination) como
prueba de selección para El diagnóstico de demência:
Estudio poblacional colombiano. Rev Neurol.

[16] Monsch AU, Bondi MW, Salmon DP (1995). Clinical validity of the Mattis Dementia Rating Scale in
detecting dementia of the Alzheimer type. A double cross-validation and
application to a community-dwelling sample. Arch Neurol.

[17] Bank AL, Yochim BP, McNeill SE, Lichtenberg PA (2000). Expanded normative data for the Mattis Dementia Rating Scale for
use with urban, elderly medical patients. Clin Neuropsychol.

[18] Rilling LM, Lucas JA, Ivnik RJ (2005). Mayo's Older African American Normative Studies: norms for the
Mattis Dementia Rating Scale. Clin Neuropsychol.

[19] Ardilla A, Bertollucci PH, Braga LW (2010). Illiteracy: the neuropsychology of cognition without
reading. Arch Clin Neuropsychol.

[20] Ostrsky-Solis F, Ardilla A, Rosselli M, Lopez-Arango G, Uriel-Mendoza V (1998). Neuropsychological test performance in illiterate
subjects. Arch Clin Neuropsychol.

[21] Bennett A, Nadler J, Spigler M, Rafalson L, Abraham S, Rilkin N (1997). The Mattis Dementia Rating Scale in nursing home octogenarians
and nonagenarians: effects of age and education. J Geratr Psychiatry Neurol.

